# Hundreds of Circular Novel Plasmids and DNA Elements Identified in a Rat Cecum Metamobilome

**DOI:** 10.1371/journal.pone.0087924

**Published:** 2014-02-04

**Authors:** Tue Sparholt Jørgensen, Zhuofei Xu, Martin Asser Hansen, Søren Johannes Sørensen, Lars Hestbjerg Hansen

**Affiliations:** 1 Department of Biology, University of Copenhagen, Copenhagen, Denmark; 2 Department of Environmental Science, Aarhus Universitet, Roskilde, Denmark; University of Illinois, United States of America

## Abstract

Metagenomic approaches are widespread in microbiological research, but so far, the knowledge on extrachromosomal DNA diversity and composition has largely remained dependant on cultivating host organisms. Even with the emergence of metagenomics, complete circular sequences are rarely identified, and have required manual curation. We propose a robust *in silico* procedure for identifying complete small plasmids in metagenomic datasets from whole genome shotgun sequencing. From one very pure and exhaustively sequenced metamobilome from rat cecum, we identified a total of 616 circular sequences, 160 of which were carrying a gene with plasmid replication domain. Further homology analyses indicated that the majority of these plasmid sequences are novel. We confirmed the circularity of the complete plasmid candidates using an inverse-type PCR approach on a subset of sequences with 95% success, confirming the existence and length of discrete sequences.

The implication of these findings is a broadened understanding of the traits of circular elements in nature and the possibility of massive data mining in existing metagenomic datasets to discover novel pools of complete plasmids thus vastly expanding the current plasmid database.

## Introduction

In 1952, Joshua Lederberg defined a plasmid as an inherited, extrachromosomal unit of genetic information and, though the definition has been refined, the term plasmid is still in use [1]. One major change to this definition was the discovery of horizontal gene transfer (HGT) and its link to plasmids. HGT in bacteria is generally accepted as a major driver of bacterial evolution and allow cells to acquire traits and sequences from neighbouring cells. The major role of plasmids in this process mean that they are now widely considered cosmopolitan inhabitants of a range of different host cells rather than strictly inherited cell features. Though plasmids are important in HGT, other elements such as transposon intermediates, bacteriophages and naked DNA are also vectors for HGT [2]–[4]. The pool of vectors of HGT that inhabit a cell is commonly referred to as its mobilome and constitute the totality of mobile genetic elements within the cell. Similarly, the communal pool of mobilomes within a niche is referred to as the metamobilome of the niche [5]–[7].

The replication systems of plasmids are diverse, not homologous and at least partly independent of chromosomal replication [8]. Plasmids that can be spread by HGT are thought to be the backbone of spread of e.g. antibiotic resistance between microorganisms, thereby being not only interesting in an evolutionary context, but also in a clinical context where they are involved in the death of thousands of humans every year. For example, it has been estimated that a single resistant bacterial species has caused the death of >18.000 humans in USA alone in one year (2005) [9].

Traditionally, a selectable trait such as an antibiotic resistance marker has been a key factor in isolation and characterizing plasmids. This slow and laborious approach have most likely also created a bias towards larger plasmids (>20,000 nucleotides (nt)) and plasmids hosted by clinically relevant species, as suggested in some recent papers [10], [11]. Contrary, plasmids with no obvious selection marker or clinical relevance have not been the focus of attention. As a result, large plasmids are dominating the NCBI plasmid database (ftp://ftp.ncbi.nih.gov/genomes/Plasmids), where the average plasmid size is 73,000 nt and the largest ones are above 1*10^6^ nt.

Small plasmids (here <20,000 nt) often do not encode known accessory functions and are termed cryptic [12]. They have long been considered genetic parasites or promoters of intragenomic recombination [13]. However, this type of plasmids could potentially be a reservoir of genetic shuffling making them a driver in bacterial evolution. They could also serve as an important source for discovery of novel replication systems.

It has been estimated that only 1% of microorganisms are culturable, and before the era of high throughput sequencing, only these cells could be examined for their extrachromosomal content. Now, technological advances have allowed the sequencing of culturable as well as unculturable bacteria, yielding many novel plasmid sequences but few complete plasmids [10], [11], [13]–[18].

The output of present day whole genome sequencing technologies are often too short for analysis, making programs for assembling reads into larger contigs an essential bioinformatic step. Many such programs have been developed with the majority relying on either the sequence overlap method (e.g. Newbler) or the de Bruijn graph method (e.g. IDBA-UD) [19], [20].

Repeated sequences generally pose an important challenge in the assembly process. That is, if a paired end (PE) read pair cannot span such a repeat, determination of the exact nucleotide sequence is not possible. With present day ultra-high throughput sequencing technology, correct and complete assembly of sequences is the most important bottleneck in many metagenomic pipelines [21]. Different methods have been used to assess the myriad of de novo assembly programs developed, but so far, there is no consensus on algorithm or program [22]. Unfortunately, no assembler to date is able to search for circular elements, which is why we developed a pipeline for post-assembly detection of circularity among contigs. As different sequencing technologies have different properties, exemplified by the short read, high throughput Illumina technology and the fewer, longer reads 454 GS FLX technology (454), different measures should be taken in assessing the output.

The sewer of a hospital in Copenhagen, Denmark has been found to carry large quantities of active and metabolized pharmaceutical compounds (e.g. antibiotics and other chemotherapeutics) along with regular human derived wastes, often from patients with infections resistant to antibacterial treatment [23]. Therefore it is believed to be a hotspot for HGT, but few species inhabiting the 37°C mammalian gastrointestinal tract will proliferate in wastewater. In contrast, the GI tract of a brown rat (*Rattus norvegicus*) inhabiting a hospital sewer is a warm and stable environment while still being exposed to potent and changing selective pressures from any wastewater chemotherapeutic compound. These features make the hospital sewer rat a suitable system for studying MGE. The brown rat is a hindgut fermenter, with a large pouch-like cecum, important for the breakdown of food that would otherwise be indigestible, primarily cellulose [24]. Inside the cecum resides a diverse microbial flora that we hypothesize is less food-dependant than in the unidirectional large intestine and therefore more stable and representative for the long-term HGT effects of chemotherapeutic exposure.

In this study we sought to demonstrate that simple sampling and processing, and standard sequencing technology, is sufficient to completely assemble a plethora of circular elements, and identify many novel plasmids within a metamobilome. Our results confirm this hypothesis and suggest that the collection of small plasmids in the NCBI genomes database is far from exhausted and not covering the diversity of for the circular genetic elements in nature.

## Materials and Methods

### Sample processing

The brown rat (*Rattus norvegicus*) cecum sample used to produce all sequences in this paper originates in a rat caught in a live trap set in the sewer of Bispebjerg Hospital in Copenhagen, Denmark. The rat was euthanized as a part of municipal pest control, and thus no animal was killed for the purposes of this study (permit not required). The cecum was excised within 30 minutes after euthanization with a pellet gun and the content was stored at –80°C in 5 volumes of PBS and 30% v/v glycerol until use. Plasmids were purified from the sample using Plasmid mini AX kit from A&A Biotechnology, Gdynia, Poland. The sample was treated as described [7]: In brief, Plasmid-safe Exonuclease (Epicenter Technologies, Madison, USA) was used to digest chromosomal DNA and a qPCR with the resultant sample was run with universal 16S primers (27f, 1492r) to confirm removal of chromosomal DNA [25]. Then, the sample was Multiple Displacement Amplified (MDA) with φ29 DNA polymerase and random hexamers (RepliG, Qiagen, Venlo, Holland), according to the manufacturers protocol. DNA was subsequently sheared with a Bioruptor from Diagenode, Liege, Belgium and the library for Illumina sequencing was constructed with NEBnext Quick DNA Library Prep Master Mix Set (NEB), followed by Agarose Gel Electrophoresis and excision of fragments between 450nt and 550 nt. Previous studies have described a similar approach to enrich plasmids [7], [26]. To generate the 100 nt PE data, a full lane on an Illumina HiSeq 2000 was run. The GS FLX Titanium Rapid Library Preparation Kit (Roche, Basel, Switzerland) was used to construct the library for 454 sequencing and here, half a plate was used to generate the 454 GS FLX data.

### Bioinformatical processing

Illumina reads were pre-processed in a UNIX environment using Biopieces (Hansen,MA, www.biopieces.org, unpublished). Adapter sequences and low quality nucleotides in the reads were detected and filtered by AdaptorRemoval [27] with default parameters. The Illumina reads were assembled *de novo* using IDBA-UD (released Oct 18, 2012*)* [19] with the following parameters: *--pre_correction --num_threads 16 --min_contig 200*. Adapter sequences in the 454 reads were detected and removed by PRINSEQ-lite 0.19.2 [28]. 454 reads with low quality were removed using Biopieces with the following parameters: *mean quality threshold of 20, local quality threshold of 15 with a 5 nt sliding window*. *De novo* assembly of 454 reads was performed using Newbler 2.6 (Roche). CLC main workbench 6.7.1 was used to visualize contigs and blast results.

BLASTS search of reads against NCBI plasmid and phage databases were performed with the following parameters: identity >95%, hit length > 89 nt, alignment length is at least 90% of the query reads. Gene finding was done with Prodigal 2.50 [29] with standard parameters, with the ‘-p meta’ switch for metagenomic sequences. The NCBI plasmid database (4000 entries) was downloaded 2013-03-10 and used for all analysis (ftp://ftp.ncbi.nih.gov/genomes/Plasmids). Complete translated gene sequences identified with Prodigal [29] was searched for functional domains using HMMER 3.0 (cut-off 10^−4^, best hit used) and the Pfam database (version 26.0) [30]. Pfam families covering known plasmid replicon domains were modified from [31] and [11] (Table 1). Alignments and neighbour joining trees were made in MEGA5 with 1000 bootstraps and visualized with Figtree [32], [33].

**Table 1:** List of Pfam families and instances among the putative plasmids.

PFAM family	Name	Description	Count
PF01446.12	Rep_1	Replication protein	47
PF01719.12	Rep_2	Plasmid replication protein	26
PF01051.16	Rep_3	Initiator Replication protein	46
PF05732.6	RepL	Firmicute plasmid replication protein	21
PF07042.6	TrfA	TrfA protein	0
PF04796.7	RepA_C	Plasmid encoded RepA protein	0
PF02486.14	Rep_trans	Replication initiation factor	5
PF01402.16	RHH_1	Ribbon-helix-helix protein, copG family	22
PF01815.11	Rop	Rop protein	2
PF03428.8	RP-C	Replication protein C N-terminal domain	0
PF10134.4	RPA	Replication initiator protein A	0
PF06970.6	RepA_N	Replication initiator protein A (RepA) N-terminus	0
PF06504.6	RepC	Replication protein C (RepC)	0
PF03090.12	Replicase	Replication initiator protein	4

Pfam families used to identify putative plasmids by replicon domain identification. On the right are instances of replicon domains from the genes found in putative plasmids. A total of 173 replicon domains were found on 160 putative plasmids.

### *In silico* detection of circular contigs

Circular DNA sequences do not have endings but assembly programs such as IDBA-UD, Velvet, SOAPdenovo and Newbler can only output linear contigs. Thus, if a circular element is sequenced with high enough coverage and assembled into a single contig, reads overlapping the ends of the contig should exist. This principle is the basis of the pipeline we developed to search for circular elements in Illumina and 454 datasets. As both the reads and the relevant assemblers have different properties between platforms, we developed one pipeline for each.

**Figure 1:**
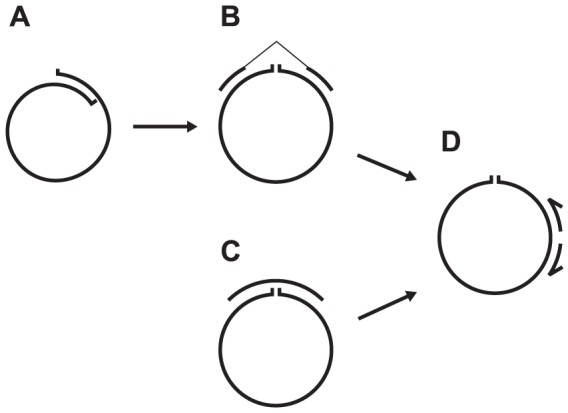
Workflow for *In silico* detection of circular contigs. A and B: *in silico* workflow for contigs from IDBA-UD (Illumina PE reads). A: detection and removal of overlapping contig ends. B: identification of read pairs that span the start/end of a contig. C: workflow for contigs from Newbler and 454 reads. A read spanning at least 100 nt on both ends of a contig is required for a contig to pass the circularity test. D: *in vitro*/inverse PCR detection of circularity. Notice that primers are located away from the start/end of the contig.

For the Illumina-IDBA-UD platform, we used the observation that contigs from circular DNA linearized by φ29 polymerase had identical ends, up to approx. 100 nt. A two-step approach was chosen, with step one being identification of identical ends (>40 nt) on contigs (Figure 1A). Positives were subjected to step two, where PE information was used to confirm this circularity by discarding all contigs where no read pairs mapped on opposite ends of a contig, a maximum 500 nt from the ends with minimum 90 nt mapping (Figure 1B). The complete pipeline with Perl scripts can be found in Information S1.

For the single end reads from the 454 platform, a different approach was taken. Here, contig ends 200nt long (100nt from each end) were extracted and a BLAST database was built from it. In the following BLASTn, if a >99 nt segment on both ends of a contig was covered by a read with no gaps and > 98%identity, the contig was put in the ‘circular’-bin (Figure 1C). To verify that the 200 nt overlap in contig in the circular bin was not due to known repeated sequences, we looked for repeats in the 4000 sequence NCBI plasmid database. None of the 200 nt sequences could be found in duplicate in any database entry, which shows that none of the ends of the circular contigs are known plasmid repetitive elements. The developed pipeline is found in Information S1.

Circular contigs from both pipelines was selected for inverse PCR confirmation of circularity (Figure 1D). The circular contigs was submitted to the EMBL and was assigned individual accession numbers in the range HG796247-HG796860 (not including the found known complete plasmids, see Table 2). The sequences are available at the European Nucleotide Archive (http://www.ebi.ac.uk/ena/data/view/HG796247-HG796860).

**Table 2:** List of known plasmids found among the putative plasmids.

Circular element/ contig (this study)	Length (nt)	Database plasmid name	Database plasmid acc. number	Database plasmid length (nt)	Replicon domain	Identity %	Reference
pRCF00019	5594	pBFP35	NC_011073	5594	Yes	99.8	[35]
pRCF00109	2101	pSS046_spC	NC_009347	2101	Yes	100	[36]
pRCI00231	5595	pBFP35	NC_011073	5594	Yes	99.8	[35]
Illumina contig00324	4900	pB80	NC_011332	4898	Yes	99.8	[37]
Illumina contig00470	4069	pIGWZ12	NC_010885	4072	Yes	99.9	[38]

Note that pRCF00019 and pRCI00231 are identical, except for a 1 nt potential sequencing error in pRCI00231 (GGG instead of GG, not in the end of the sequence).

### Primer design and PCR

Primers for inverse PCR were designed using the Primer3 web interface version 0.4.0 [34] with standard parameters except: Max Repeat Mispriming = 25, Pair Max Repeat Mispriming = 50, Max Template Mispriming = 25, Pair Max Template Mispriming = 50. Mispriming was then investigated by virtual PCR using the Biopieces function ‘pcr_seq’. All contigs >200 nt from assembly of Illumina and 454 reads were loaded twice to mimic circularity and used as mispriming database. Following criteria was used: 1 mismatch allowed, 1 deletion allowed, 1 insertion allowed. All primers used passed this mispriming test. Primers were obtained from TAGC (Copenhagen, Denmark). PCRs were run with Phusion hot-start polymerase (Thermo-Fischer Scientific, Waltham, Massachusetts), according to protocol. Annealing temperatures varied between 56 and 61.5°C depending on T_m_ calculated by the Primer3 web interface [34] (see Table S1 for list of primers used). Four minutes elongation and 35 cycles were used in all cases. All PCRs were run at least twice. 3% DMSO was used if no product materialized without it. As template, a 100 fold dilution of the original MDA sample was used corresponding to 1,46ng DNA per reaction. PCR products were visualized on 1% agarose gels with ethidium bromide post staining.

## Results and Discussion

### Sequencing output

The output from the Illumina platform was 161,902,848 reads totalling 1.6*10^10^ nt. In the trimming and filtering process, 1.3% of these were removed leaving 159,866,488 reads. The total number of nucleotides after filtering was 1.5*10^10^ nt. For 454 sequencing, the total output was 292,811 reads with 1.2*10^8^ nt. 5.5% of the reads was removed in the filtering process with 276,656 reads left (1.1*10^8^ nt). The chromosomal contamination was investigated and for either platform, <3% of reads was expected to be of chromosomal origin. For a detailed characterization of the purity and composition of the reads see Information S2.

The assembly statistics from the two platforms are comparable in categories such as average contig length, maximum contig length and N50, but in the categories cumulative contig length and number of contigs, the output from the Illumina platform is more than 10-fold greater than from the 454 platform (Information S2).

**Figure 2:**
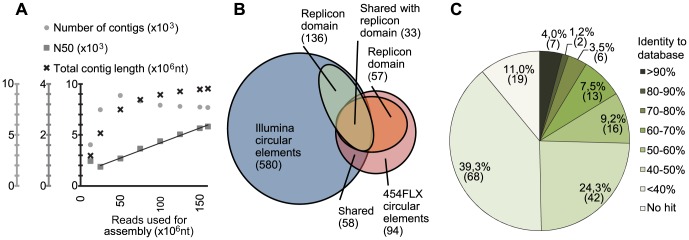
Rarefaction plot and circular elements composition. A, rarefaction estimation of sequencing depth by assembly of random subsets of Illumina PE read pairs. Crosses show total contig length as a function of subset size. The saturation that can be seen after the 50(R^2^  =  0.99), suggesting that information on assembly of contigs are not exhausted. Circles show contig number as a function of reads used for assembly. Notably, fewer contigs are found with larger subset sizes, suggesting that contigs have been joined as little total contig length s added. B, Diagram of circular sequences found in the Illumina and 454 dataset. In total, 616 sequences were found to be circular. Of these, 160 sequences had 173 predicted genes with replicon domains. C, identity between replicon domain genes from putative plasmids and the best BLAST hit in the plasmid database. Binning of the BLAST hits by identity percent reveals that few replicon domain genes are similar to database versions while many are very different from database versions. Thus, 50.3% of replicon domain genes have less than 40% sequence identity to database version while 8.7% of replicon domain genes in the putative plasmids have more than 70% identity.

In order to estimate how exhaustively the mobilome was sequenced with the Illumina platform, we did *de novo* assembly of random subsets of the Illumina dataset using IDBA-UD (Information S3). We found that the full 160 M reads dataset gave a solid representation of the DNA sequences present in the sample, in that the increase in total contig length was negligible after the first approx. 57 M reads. Observing the number of contigs shows an initial increase until 50 M reads and interestingly a slight but steady drop thereafter. This indicates that many sequences are completely assembled and thus ‘assembly inert’, as would be the case for a mobilome sample containing only small circular starting material. Also interesting was the steady increase of N50, an indication that the information on how reads should be assembled was not exhausted by the full 160 M reads dataset (Figure 2A).

### Detection of circularity and biases

The two-step process of identifying circular elements from the Illumina platform assembled with IDBA-UD (Figure 1 A and B) yielded 580 putative circular sequences. The minimum size of circular contigs was set to 1000 nt to avoid overlaps of sequence windows used in step two of the circularity detection. The largest of the sequences was 12,541 nt and the average length was 2,434 nt, with contigs ranging in coverage between 7.8 and 55,589.8. The one-step identification of circular elements from the 454 platform (Figure 1C) yielded 94 sequences. These range up to 8,935 nt with an average of 3,105 nt. 58 sequences were detected in both datasets (Figure 2B). The Illumina versions were removed prior to analysis. Of the 36 circular contigs found in the 454 dataset and not in the Illumina dataset, all had Illumina contigs of equivalent length and sequence that was not picked up as circular (data not shown). This difference might be caused by the different properties of the sequencing platforms or assembly process.

The size distribution in the detected circular elements is very different from the NCBI plasmid database, where the average length is 78,000 nt. A number of factors are contributing to this difference: 1, favoured amplification of small circular elements by φ29 polymerase. 2, the size distribution of database plasmids may not represent the size distribution of plasmids in nature, an explanation also suggested previously [10], [11]. 3, in the sequencing and assembly process, a short circular element will have a smaller risk of coverage insufficiency than longer circular elements with the same coverage. 4, the inability of IDBA-UD to assemble sequences with repeats longer than a read. As many large plasmids are infested with repeats of varying type, length and origin (e.g. IS elements), these will not be assembled to single contigs and thus remain undetected regardless of sequencing depth. 5, larger plasmids are more likely to shear during the plasmid purification and subsequently be broken down by exonucleases prior to sequencing. 6, the copy number of small plasmids is often very high compared to larger plasmids, leading to a smaller risk of coverage insufficiency. 7, very large plasmids >1 Mb exist in the database skewing the average length.

To investigate to which extent the identified circular elements were already annotated in the NCBI plasmid database, we BLAST searched contigs against the NCBI plasmid database. Five contigs could be identified as complete known plasmids; one circular from both platforms, one circular solely from the 454 platform and two non-circular Illumina contigs that were not picked up by our circularity detection (Table 2)[35]–[38]. The explanation is that these two contigs did not pass step one of the circularity detection (Figure 1A), where a gap/misassembly of even a few nucleotides will not be tolerated as it will lead to contig ends not overlapping. This conservative approach will lead to false negative results, but will not lead to false positive results. That only two known plasmids are found in the pool of hundreds of circular elements found is surprising and points to a database underrepresentation of small circular elements. Besides the complete plasmids, a number of fragments were found in the above BLAST search, possibly reflecting shared features between database plasmids and the circular elements identified in this study (data not shown).

### Replicon domain Pfams

In order to identify plasmid-like sequences within the circular elements, we chose to focus on complete predicted genes with plasmid replicon domains, a domain commonly used to classify plasmids [8], [39], [40]. From the Illumina dataset, 129 putative plasmids were found to contain replicon domain (22%) and from the 454 dataset, 64 out of 94 circular sequences were found to encode a replicon domain (68%). The difference can largely be assigned the different sequencing depth of the datasets as 50% of the elements with highest coverage from the Illumina platform were found to harbour a replicon domain. Similarly searched using Pfam, 75% of plasmids in the NCBI plasmid database encode a replicon domain. Further, a plasmid such as ColE1 does not use a replication initiation protein at all, but an RNA initiator and would not be identified by our replicon domain search [41]. Finally, it is very likely that that some circular elements encode yet unknown replication systems, particularly as the bacteria contributing to the mobilome were not cultured.

**Figure 3:**
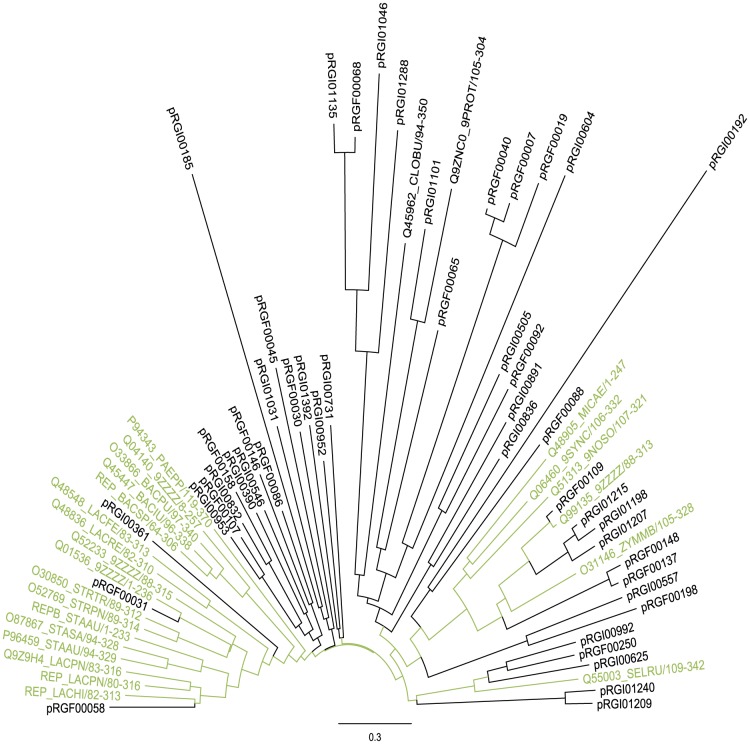
Phylogenetic tree of novel and known Rep_1 sequences. Phylogenetic neighbour joining tree of Rep_1 sequences from translated complete genes from putative plasmids (black) and Pfam seed sequences (green). Many diverging clades and sequences are seen, reflecting the diversity of replication proteins in the natural environment.

To explore nucleotide level similarity between the predicted replication genes and known versions, we BLAST searched the genes against the NCBI nr/nt database. A summation of the best hits can be seen in Figure 2C. Out of 173 predicted genes encoding a replicon domain (160 contigs), 4.0% (7) share more than 90% Identity with the known plasmids on a nucleotide level. Contrary, 74.6% (129) showed less than 50% identity at the nucleotide level to a known version (Figure 2C). On this basis, we conclude that the majority of replicon domains found in putative plasmids is not closely related to any database version. To achieve a graphical phylogenetic overview of the identified replication genes, we created a neighbour joining tree for each replication protein family. Translated genes from putative plasmids were trimmed to the length of the relevant Pfam seed database of representative sequences. This seed database was then used to visualize the diversity of database versions compared to versions identified in this study. As exemplified by the Rep_1 neighbour joining tree in Figure 3, many hitherto unknown branches and deep-branching clades and can be seen, demonstrating the addition of diversity to the seed database by sequences found in this study. The remaining seven neighbour joining trees can be found in Figure S1.

The finding of significantly different replication proteins is important as it greatly expands the known groups of replication proteins and opens for a broader understanding of the diversity of sequences within the replication protein families.

Besides plasmid replication domains, some of the 616 circular sequences were found to harbour other plasmid-like traits such as genes with toxin (4), antitoxin(2), Mob(50) and T4SS(5) domains, all expected to be found in a plasmid pool. Some genes with primarily phage-related domains were also observed, with capsid (21) and phage integrase (83) being the most prominent ones (number of complete predicted gene PFAM domains in parenthesis). 98 complete genes with transposase domain was also found, possibly owing to circular intermediates of certain IS elements [42].

### Validation of circularity

In order to test if the contigs *in silico* identified to be circular could be amplified *in vitro*, we designed primers for an inverse PCR type approach [43]. As there is very limited possibility of repeats in the assembly (described in Information S4), primers pointing away from each other with no gap between them can only yield a product if the template is circular, or as in this case, multiplied and subsequently linearized by MDA. By applying this approach directly to the MDA sample, we were able to recreate the complete sequence of selected contigs, thereby not only confirming their existence, but also their size in one PCR.

We chose to test 40 of the 616 circular elements with inverse PCR. Sequences to be tested were equally distributed on sequencing platforms (28 from each platform, including 16 found in both datasets) and both sequences with and without a replicon domain were tested (34 with, 6 without). Coverage or other parameters that might bias the outcome were not considered. Four of the tested circular elements were found to encode a gene with either a transposase-like Pfam domain or an integrase-like Pfam domain. For four elements, gap closing primers were tested and in all cases produced a product of the expected length (data not shown).

**Figure 4:**
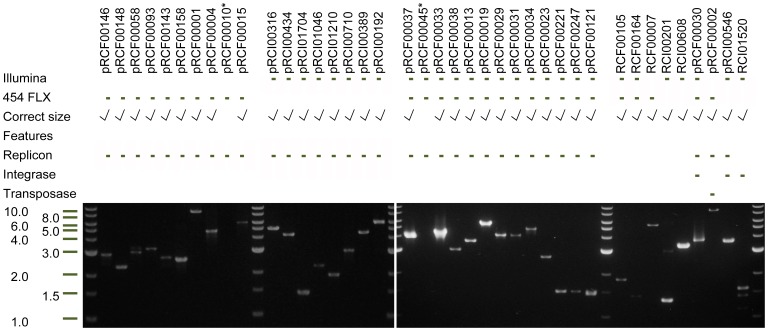
Inverse PCR confirmation of circularity. PCR results from primers targeting 40 sequences, 28 from 454 and 28 from Illumina. Of these, only 2 fail to produce a product of the expected size (marked with *). Further, secondary products are only seen in two cases (RCI00201 and RCI01520). Of the 40 circular elements tested, 34 encode a replicon domain; three encode an integrase domain, and one a transposase domain. Of five sequences with none of the traits, all produce a product with the expected length.

Of the 40 circular elements, 38 yielded a product of the expected size and only two of those produced notable secondary products (Figure 4). This overall rate of success of 95% is a strong indication that the contigs are in fact entire circular elements. The fact that only two contigs yielded more than one band is also an indication of the high specificity of primers and the discreteness of the individual circular element sequences. For sequences encoding a replicon domain, 32 out of 34 produced a band of the expected length.

The very high rate of success across sequencing platforms and encoded traits suggest that the results can be extrapolated to the entire group of circular elements from both platforms, a total of 616 sequences; 94 from 454, 522 from Illumina (58 duplicates removed) including two already known plasmids. This implies that the *in silico* search for circular elements presented here can often stand alone with no manual curation of sequences or *in vitro* test of results, and thereby constitute a powerful and easy tool for data mining existing of datasets as well as new sequencing projects for complete, circular sequences.

A recent article [11] investigating sea metagenomes found many small contigs that have plasmid-like traits and the authors speculate that the contigs are complete circular plasmids. Likewise, an inverse PCR approach with degenerate primers was used to amplify small, dominating Rep_3-like contigs. On this basis, the authors have proposed that small plasmids are more abundant in nature than in the NCBI plasmid database. This is very much in line with our findings and explains how more than 100 new plasmids can be identified in a single mobilome. We here move one step further, using both overlap and paired end information to *in silico* predict complete plasmids. The same approach is taken in a recent study of vira [44]. Here, 60 complete ssDNA vira are extracted from ultrafiltered human stool samples. Their circularization approach is contig overlap search using Minimo (http://sourceforge.net/apps/mediawiki/amos/index.php?title=Minimo), but they do not take into consideration paired end information to further establish the circular nature of the identified sequences.

In another recent study [14], complete plasmids up to 59,000 nt were recovered from plasmidomes/mobilomes obtained by CsCl-Ethidium Bromide density gradient centrifugation of 5 L sludge samples from wastewater plants. Here, the sequence and circularity of scaffolds built from assembly were determined by PCR. They, as well as Ma et al. [11] find novel replicon domains in the sample, supporting the findings in this paper. The study was the first to extract many complete plasmid sequences from a metagenome-type sample [14]. However, the large sample volume required, tedious plasmid purification and manual finishing of plasmids (closing gaps with Sanger sequencing) make the method unlikely to be used widely in metagenomic projects. Opposed to this, the method presented here requires only standard laboratory equipment, no manual curation and a small initial sample, in this case less than one gram of rat cecum. For future research to achieve an unbiased overview of plasmids and circular elements in nature, a key element is the sequencing technology, as present-day short read technology seems unable to overcome repeated structures on circular elements.

## Conclusions

In this study, we have identified an immense pool of unknown circular extrachromosomal units from a single metamobilome sample from a single rat cecum. We show that the results from the *in silico* plasmid identification can be confirmed *in vitro* with a very high success rate (95%) and little hazard of false positives as a result of interspersed repeats for the Illumina platform.

The 160 plasmids identified in this study constitute a 40% increase in the NCBI plasmid database in the size range 1,000–13,000 nt. In addition to this, 456 circular elements were found, that are not confirmed to be plasmids. This addition of plasmids stem from a single sample, indicating that the entries in the NCBI plasmid database is far from exhausting and possibly not representative for the diversity of plasmids in nature. We expect that data mining and future metamobilome studies will lead to a database better reflecting the composition of plasmids in natural environments, instead of being focused on clinically relevant plasmids or plasmids with culturable hosts.

Hopefully, future research will explore of the world of small plasmids and move many of them from the ‘cryptic’ category to more biologically meaningful categories.

## Supporting Information

Figure S1 **Phylogenetic trees of translated replicon genes and PFAM seed sequences.**
(PDF)

Table S1 **List of primers used.**
(DOCX)

Information S1 **Procedure for automated circularization of contigs.**
(DOCX)

Information S2 **Estimation of chromosomal contamination and Sequencing output composition.**
(DOCX)

Information S3 **Rarefaction type evaluation of sequencing depth.**
(DOCX)

Information S4 **Test of assembly of sequences with interspersed repeats using IDBA-UD.**
(DOCX)
