# Supplementary figures and images for: Hundreds of Circular Novel Plasmids and DNA Elements Identified in a Rat Cecum Metamobilome
Source: PLoS One. 2014 Feb 4;9(2):e87924. doi: 10.1371/journal.pone.0087924 (PMC3913684; doi:10.1371/journal.pone.0087924)

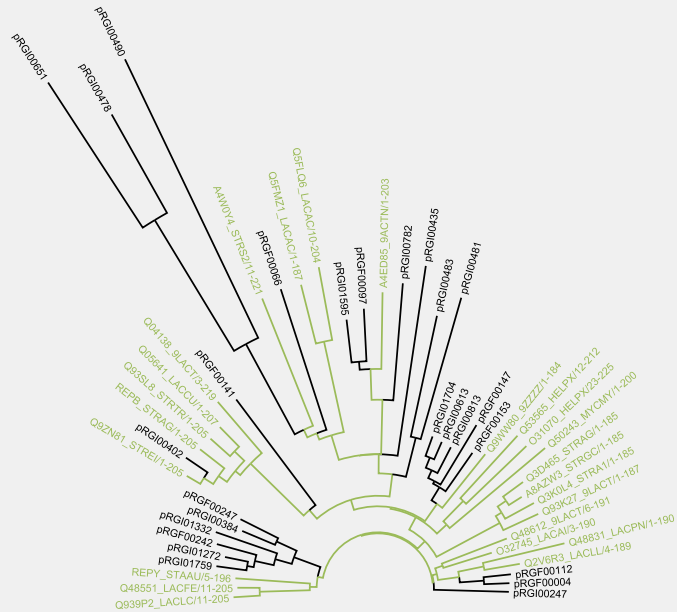

Rep\_3

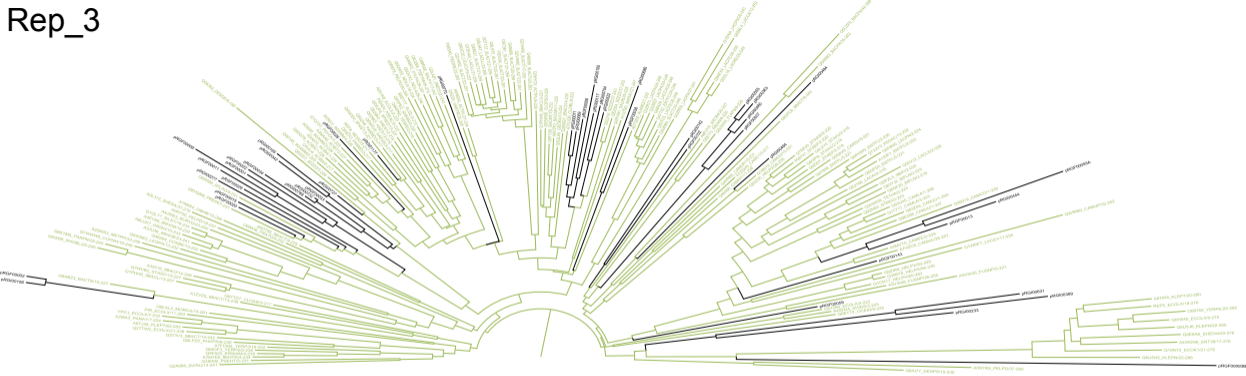

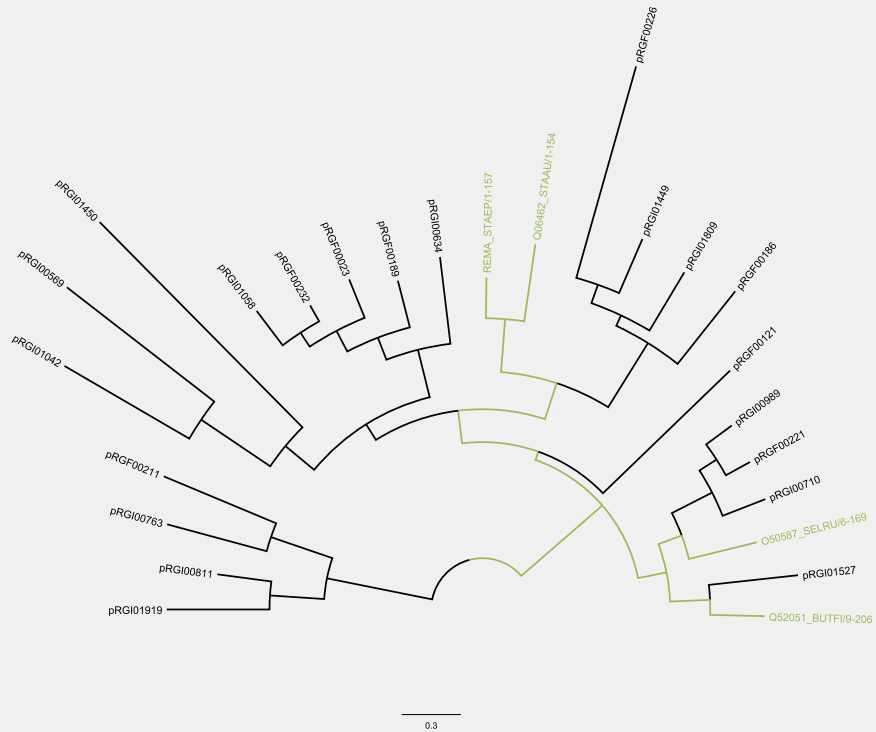

## Rep\_trans

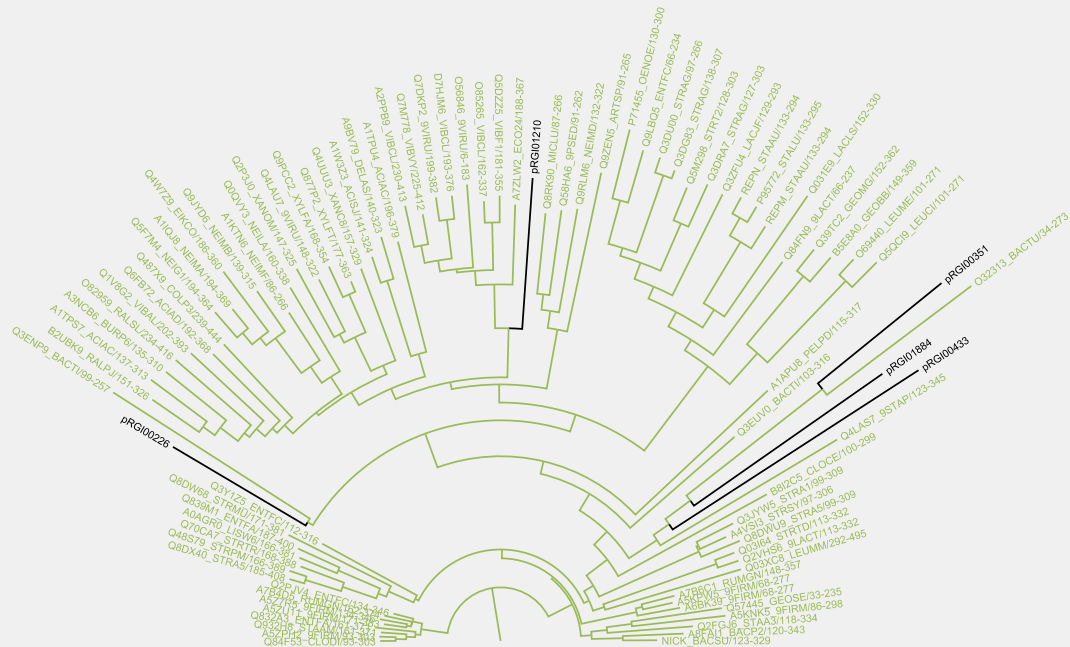

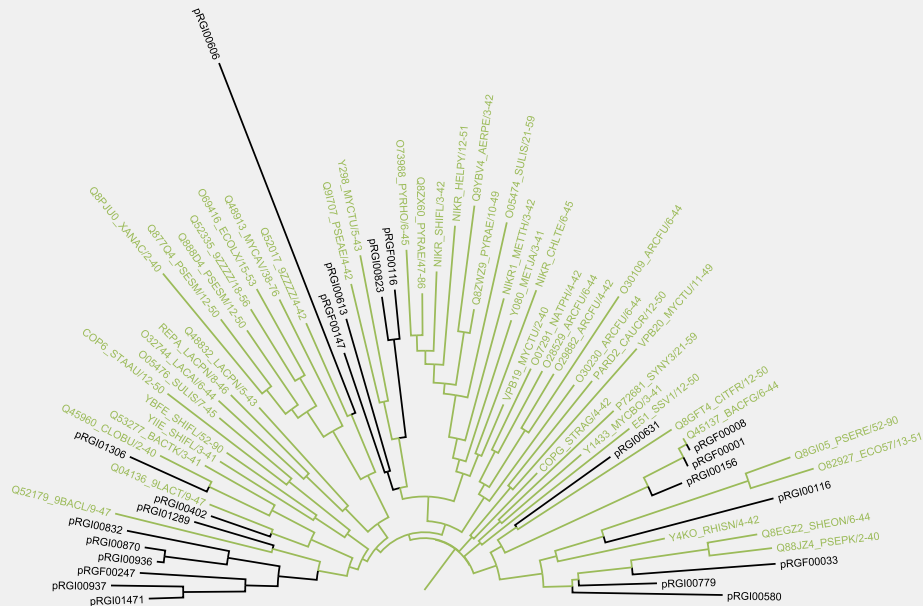

Rop

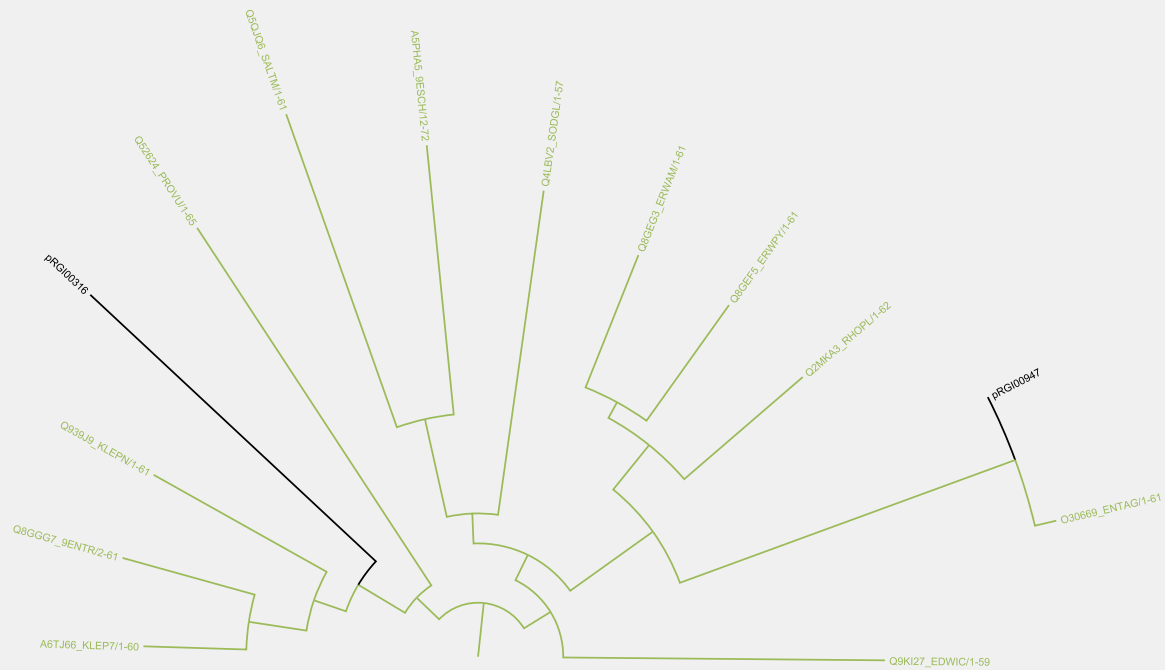

0.05

# Replicase

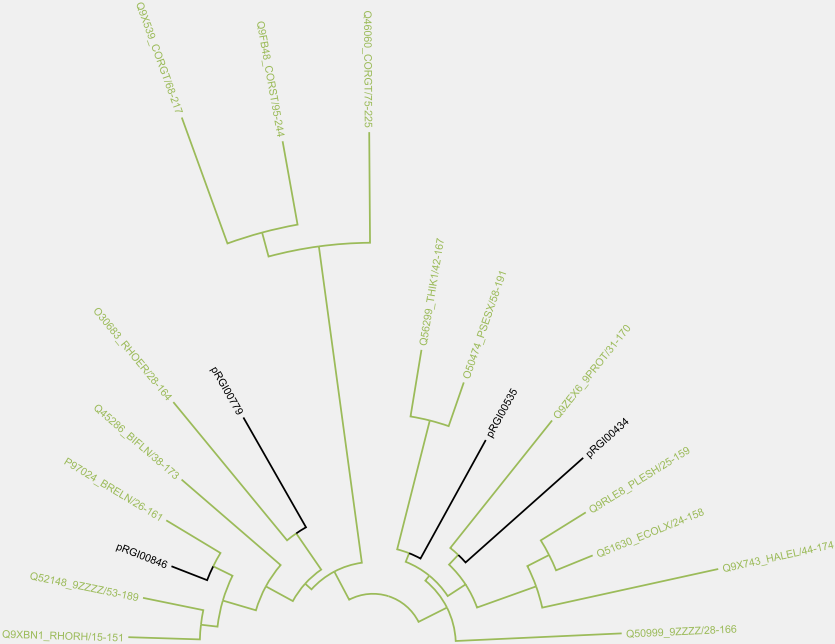

Supplement: Figure S1 — Phylogenetic trees of translated replicon genes and PFAM seed sequences. (PDF) [file pone.0087924.s001.pdf]
