# Supplementary material for: Hundreds of Circular Novel Plasmids and DNA Elements Identified in a Rat Cecum Metamobilome
Source: PLoS One. 2014 Feb 4;9(2):e87924. doi: 10.1371/journal.pone.0087924 (PMC3913684; doi:10.1371/journal.pone.0087924)
Supplement: Table S1 — List of primers used. (DOCX) [file pone.0087924.s002.docx]

**Supporting table S1. List of primers used.**

| circular element name | forward primer (5’ - 3’) | reverse primer (5’ - 3’) |
| --- | --- | --- |
| pRCF00001 | GAGCCCGGGGAGTCAATATTTTACGCGGAT | CTCCCCGGGCTCTAACTGTTATATGATGATTAAATTATC |
| pRCF00002 | GAGCCCGGGGAGAGGAGTTTGTTATGAATGAAAATA | CTCCCCGGGCTCTTATTTGTAACTGCTTTTTATACCA |
| pRCF00004 | GAGCCCGGGGAGCTTTAAGTGCTGTGTTCGTCA | CTCCCCGGGCTCTTACCTACGTACAATACCTCTTTGA |
| pRCF00007 | GAGCCCGGGGAGTTATATATGTAATCCTTTACCAATATCA | GAGCTCGAGGAGAAAAGAATGGGAGCATTAAA |
| pRCF00010 | GAGCCCGGGGAGTGAATAAATTGCCTCAATAAAATTT | CTCCCCGGGCTCATGGACGCATACAACACAG |
| pRCF00013 | CGACACACAAGCACGAAAAG | GCTTCGGTTTGTATGGCAAC |
| pRCF00015 | ATAATCTCGGCAAGCAGCTC | CAACAGTTCCGACAACAACG |
| pRCF00019 | GAGCCCGGGGAGAATAGTGGGTAGTTGTTAGTTTGTT | CTCCCCGGGCTCAGGAAAAATATCCTCATCTAAATAAA |
| pRCF00023 | TAGTCACAGCCCCGAAAGAC | CCCAAGACACCATCACATTG |
| pRCF00029 | TGAAGGTTGAGGGAATGAGG | GACCGTAACCAACCGCTAAA |
| pRCF00030 | CCGACCAAATTACCAACTCAA | GTGTCGCAGCCCCATTATAC |
| pRCF00031 | TTTATCAATGCGCTGAGGTG | CCCGTTCAAAAGAGCGATAG |
| pRCF00033 | AATTTCTGCCGTGACCTTGT | TTGTATCAGGGTTCGGCTCT |
| pRCF00034 | CGGTCTTCGTAGGACAGGAA | AAACCCCTGCTTGCGTATAG |
| pRCF00037 | TGCCCAATATGTCTGGAACA | TAGGGAAAGACCCGGAAAAC |
| pRCF00038 | ACAGCTGAAAGGAGGTGTGC | AGGAGGATGACAGCAACGAG |
| pRCF00045 | CCATTGCATTCCCTAGCACT | GAACGATTGCCACGATTATG |
| pRCF00058 | TGTGGCAAAAAGCAATGAAA | AACTGCGCCACTCTTTTTGT |
| pRCF00093 | AAAAGGCGTTCATTCTCTGC | ACAAGCAGTGCAAACGCTTA |
| RCF00105 | TTGATAAAGCCCGCAGAGAG | TATGCTGTGCTTGTCCCATC |
| pRCF00121 | CTCTGAAAGAATCCCGAAGG | ACTTTTCCGCTTTTGCCTTT |
| pRCF00143 | CCATTGAGCCTGTCCGTAAT | CAGCGAAAATCAGAAACGTG |
| pRCF00146 | TGCCGGTCTTATGGTGGTAT | AGTCAAGATGAGCTGCACGA |
| pRCF00148 | GTCGCATATGTGAAGCAAGC | ACCGGAAAAATACCATGACG |
| pRCF00158 | TTTTGATGCTTGGGGTAAGC | AGCCGTTGTACGGTCTCTGT |
| RCF00164 | TCGCATATGTAGGAAAATCGAA | AGCGTTTTGGTTGGGTTTC |
| pRCF00221 | TTTGGATGTGTTGCAGGAAG | GAGCGAGAACGCAGTCAAG |
| pRCF00247 | CACGGCTGACGTTCGTAGTA | TCATATTGCGCTTTTTCTGG |
| pRCI00192 | GAGCCCGGGGAGTTAAAATGGCGCTTCTTC | GAGCTCGAGGAGCCAAATATAAAGCCCCTG |
| RCI00201 | GAGCCCGGGGAGGGTGTATTTTAATCACCCTAATCT | GAGCTCGAGGAGCTAATCTTGAAAGTAATAGCATTTATC |
| pRCI00316 | GAGCCCGGGGAGTCATCTTTTAGTCGCAAGTG | GAGCTCGAGGAGAAAAAAAGCACCTGAAAGG |
| pRCI00389 | CGTCAAGCCAAGTCTCTTCC | ACAGGGTGAAGCCTGTCTGT |
| pRCI00434 | GAGCCCGGGGAGTCATTTTTCACCTCCTATAAAAC | GAGCTCGAGGAGGAAGAAGCACAATAAGAAAAGGT |
| pRCI00546 | GCTTTCGCTTCCAAAAATCA | GACCAAAATCGGGGTCTTTT |
| RCI00608 | CGGTATAATGCCTTGCGAAC | GGTAGTGGTTCCTGCCTTGA |
| pRCI00710 | GCCTTGTGCCGAGTGAGTAT | ACTCGCTCCGTTATTCCAAG |
| pRCI01046 | CGAGGAGGTGAACCACAAGT | ATTGGCAGCGTCAAGAATTT |
| pRCI01210 | ATCACCGCCTTGTCTTTTTG | CTTCCGGGTCACGATTAGAC |
| RCI01520 | AACGAGGAACGTCCTCACAT | GTAGAAGCGTACCGCAGAGG |
| pRCI01704 | GCCCTTTGCATGATATGGAT | TAATGGCACATTGCAAACCA |
