# Supplementary material for: Hundreds of Circular Novel Plasmids and DNA Elements Identified in a Rat Cecum Metamobilome
Source: PLoS One. 2014 Feb 4;9(2):e87924. doi: 10.1371/journal.pone.0087924 (PMC3913684; doi:10.1371/journal.pone.0087924)
Supplement: Information S1 — Procedure for automated circularization of contigs. (DOCX) [file pone.0087924.s003.docx]

**Supporting Information S1. Procedure for automated circularization of contigs.**

**For Illumina data**

**Identification of circular contigs using overlapping sequences at both ends and information of paired-end reads**

**Design principle**

If the contigs assembled by IDBA-UD is circular, it should meet the following 2 principles simultaneously:

1) Overlapping sequences might be present in both ends of the contig. IDBA-UD doesn’t automatically cut such sequences.

2) We should find some paired reads located on both ends, respectively.

**Test data**

1 contig: Contig00032.fna (**/home/zfxu/data/tue/testdata/Illumina**)

Clean reads in FASTA format: read.fa

Script path: */home/zfxu/install/perl/*

====Requirement=======

=====================

(1)   Unix

-          The program has only been tested on UNIX

(2)   BLAST

-          blastall 2.2.22

(3)   Package AMOS (<http://sourceforge.net/projects/amos/files/>)

(4)   Perl

-          Standard Perl

-          Bioperl

**First step.**

**Identify circular contigs using overlapping sequences in both ends of the contigs.**

Chop one contig in half at the middle of the sequence and then merge overlapping sequences through the program minimus2 in the package AMOS. If you get one contig back, you now have a perfect linear representation of a circular contig.

Chop one contig in half at the middle of the sequence. It will generate 2 sequences per contig

*perl /home/zfxu/install/perl/chop.sequence.pl Contig00032.fna*

Merge overlapping sequences using minimus2 with default parameters:

*perl /home/zfxu/install/perl/minimus2.pl chopContig > nohup.minimus2.out*

Put all the resulting *.fasta files into a new folder *fasta*. Overlapping sequences at the previous ends have been removed.

*mkdir fasta*

*cp chopContig/*.fasta fasta/*

Put all detected circular contigs into a file

*perl /home/zfxu/install/perl/collection.pl fasta > circular.fna*

Optional: Extract contigs more than 1kb (the majority of true plasmids should be more than 1kb). Please install Biopieces (<http://code.google.com/p/biopieces/>) if you want to run the following extraction.

*read_fasta -i circular.fna | grab -e 'SEQ_LEN>=1000' | write_fasta -x -o contig1kb.fna*

**Second step**

**Use paired-end information to further determine the circular contig**

Hypothesis: If the contig is circular, we should find some read pairs to connect both ends. Please use the original contigs to do analysis not ones from the first step!

Extract both ends (500 bp) of the original contig (more than 1kb)

*perl /home/zfxu/install/perl/fasta_manipulate.Endseq.pl Contig00032.fna 500*

It will generate 2 files: contig.leftseq.fa and contig.rightseq.fa

The name of raw Illumina PE reads need to be modified: remove blank and add length values to the ends of the head line!

*perl /home/zfxu/install/perl/fasta_manipulate.PEheader.pl read.fa > read.name.fa*

Preparation of 2 databases for blastn

*formatdb -i contig.leftseq.fa -p F -o T*

*formatdb -i contig.rightseq.fa -p F -o T*

*blastall -p blastn -d contig.leftseq.fa -i read.name.fa -o read2contigLeft.blastn -e 1e-10 -F F -m 8*

*blastall -p blastn -d contig.rightseq.fa -i read.name.fa -o read2contigRight.blastn -e 1e-10 -F F -m 8*

To define the number of Illumina short reads (100 bp, 150 bp, 250 bp) matched to each end of each contigs, we used the following criteria: 100% alignment identity, alignment length of hit read at least 90 bp, and alignment length accounting for the hit read sequence should be greater than 99%.

*perl /home/zfxu/install/perl/parse_PEread.1.pl read2contigRight.blastn > hitread2Right.tab*

*perl /home/zfxu/install/perl/parse_PEread.1.pl read2contigLeft.blastn > hitread2Left.tab*

For the left end of the contig, only reserve the reads that are complementary reverse to the contig sequence.

*perl /home/zfxu/install/perl/parse_PEread.2.pl hitread2Left.tab > hitread2Left.reverse.tab*

If one of a read pair located on the left of the contig, check the other one of a read pair whether located on the right of the contig or not. If true, it will result in 2 files: the contig name (circular.PE.list) and the corresponding read names (circular.PE.read.list).

*perl /home/zfxu/install/perl/parse_PEread.3.pl hitread2Left.reverse.tab hitread2Right.tab*

Remove the duplicated contig names and reserve only one per contig

*sort circular.PE.list | uniq > circular.PE.sort.list*

**Use the intersection of 2 tests as final result**

*perl /home/zfxu/install/perl/intesection.pl step1/circular.fna step2/circular.PE.sort.list > circular.final.list*

**For 454 data**

**Identification of circular contigs using information of single end reads**

**Design principle**

If the contigs assembled by newbler is circular, it should meet the following principle:

1) The contigs should not have overlapping sequences in both ends. We should find some reads (mean length > 400 bp) just across both ends and perfect matching.

**Test data**

1 contig: contig.fna (/home/zfxu/data/tue/testdata/454)

Some reads: read.fa

Extract 100bp from each end of contigs, respectively. Concatenate two 100-bp fragments, put the right fragment ahead, and then followed by the left fragment.

*perl /home/zfxu/install/perl/fasta_manipulate.pl contig.fna > 200bpEnd.fas*

To detect reads that can entirely match the concatenation of both ends of a contig, a *blastn* search using all reads need to be performed against all 200-bp concatenated sequences per contig.

*formatdb -i 200bpEnd.fas -p F*

*blastall -p blastn -d 200bpEnd.fas -i* read.fa *-o read2Ends.m8.blastn -F F -e 1e-10 -m 8*

Criteria for extracting hit: alignment length 200bp without gap, 99% identity

*perl /home/zfxu/install/perl/extractHit454.1.pl read2Ends.m8.blastn > hit.m8.blastn*

The list of contigs detected to be putative circular sequence

*perl /home/zfxu/install/perl/extractHit454.2.pl hit.m8.blastn > circularContig.list*

**Nomenclature**

We named the circularised contigs reflecting the rat gut origin (*Rattus Cecum*, RC) and sequencing platform used (Illumina, I or 454 FLX, F ), followed by contig number. If a replicon domain is found, a ‘p’ precedes the name to denote that the circular element has been identified as a plasmid. For example, contig00033 from the 454 platform is named pRCF00033. As 58 contigs are shared between the two platforms (see below), we removed these duplicates from the Illumina contigs before naming and analysis.

scripts

| Script name | Script detail |
| --- | --- |
| chop.sequence.pl | #!/usr/bin/perl -w  use strict;  #Zhuofei Xu, April 03, 2013  die "Usage: perl $0 contig.fna \n" if(@ARGV != 1);  my $dir = "./"."chopContig";  system("mkdir chopContig");  use Bio::SeqIO;  my $input = shift @ARGV;  my $in = new Bio::SeqIO(-format => 'fasta', -file => "$input");  my $count = 0;  my $len = 0;  my $middle = 0;  my $left = '';  my $right = '';  my $name = '';  while( my $seq = $in->next_seq ) {  $count++;  $name = $seq->id;  $len = $seq->length;  $middle = int($len/2);  $left = $seq->subseq(1,$middle);  $right = $seq->subseq(($middle+1),$len);  open (OUT, ">$dir/$name.fa")\|\| die "can't open file:$!\n";  print OUT ">$name.left.sequence\n$left\n>$name.right.sequence\n$right\n";  } |
| minimus2.pl | #!/usr/bin/perl  #Zhuofei Xu, April 03, 2013  die "Usage: $0 circularContig" unless (@ARGV == 1);  my $dir = "./"."$ARGV[0]";  opendir(DIR, $dir) \|\| die "Can't open directory $dir\n";  @array = ();  @array = readdir(DIR);  my $name = '';  foreach my $file (@array){  next unless ($file =~ /^\S+.fa$/);  if ($file =~ /^(\S+).fa$/){  $name = $1;  }  system ("/usr/local/src/amos-3.1.0-rc1/bin/toAmos -s $dir/$name.fa -o $dir/$name.afg");  system ("/usr/local/src/amos-3.1.0-rc1/bin/bank-transact -c -z -b $dir/$name.bnk -m $dir/$name.afg");  system ("/usr/local/src/amos-3.1.0-rc1/bin/dumpreads $dir/$name.bnk -M 1 > $dir/$name.ref.seq");  system ("/usr/local/src/amos-3.1.0-rc1/bin/dumpreads $dir/$name.bnk -m 1 > $dir/$name.qry.seq");  system ("/usr/local/bin/nucmer -maxmatch -c 40 $dir/$name.ref.seq $dir/$name.qry.seq -p $name");  system ("mv $name.delta $dir/");  system ("/usr/local/bin/show-coords -H -c -l -o -r -I 94 $dir/$name.delta \| /usr/local/src/amos-3.1.0-rc1/bin/nucmerAnnotate \| egrep 'BEGIN\|END\|CONTAIN\|IDENTITY' > $dir/$name.coords");  system ("/usr/local/src/amos-3.1.0-rc1/bin/nucmer2ovl -ignore 20 -tab $dir/$name.coords \| /usr/local/src/amos-3.1.0-rc1/bin/sort2 > $dir/$name.ovl");  system ("/usr/local/src/amos-3.1.0-rc1/bin/ovl2OVL $dir/$name.ovl > $dir/$name.OVL");  system ("rm -f $dir/$name.bnk/OVL.*");  system ("/usr/local/src/amos-3.1.0-rc1/bin/bank-transact -z -b $dir/$name.bnk -m $dir/$name.OVL");  system ("/usr/local/src/amos-3.1.0-rc1/bin/tigger -b $dir/$name.bnk");  system ("/usr/local/src/amos-3.1.0-rc1/bin/make-consensus -B -e 0.06 -b $dir/$name.bnk -w 15");  system ("/usr/local/src/amos-3.1.0-rc1/bin/bank2contig $dir/$name.bnk > $dir/$name.contig");  system ("/usr/local/src/amos-3.1.0-rc1/bin/bank2fasta -b $dir/$name.bnk > $dir/$name.fasta");  }  closedir (DIR); |
| collection.pl | #!/usr/bin/perl  #Zhuofei Xu, April 03, 2013  die "Usage: $0 circularContig(Dir) > circularcontig.fna" unless (@ARGV == 1);  my $dir = "./"."$ARGV[0]";  opendir(DIR, $dir) \|\| die "Can't open directory $dir\n";  @array = ();  @array = readdir(DIR);  my $name = '';  foreach my $file (@array){  next unless ($file =~ /^\S+.fasta$/);  if ($file =~ /^(\S+).fasta$/){  $name = $1;  }  open (FILE, "$dir/$file")\|\|die "can't open file:$!\n";  while(<FILE>){  chomp;  if (/^$/){next;}  if(/^>/){  print ">$name\n";  }elsif(/^\S+$/){  print "$_\n";  }  }  close FILE;  }  closedir (DIR); |
| fasta_manipulate.Endseq.pl | #! user/bin/perl  #Zhuofei Xu, 2012-09-11  die "Usage: perl $0 fasta.input (sequence in multi-line) extractLength> out\n" unless (@ARGV == 2);  open (FILE, "$ARGV[0]")\|\|die "cannot open $ARGV[0]\n";  open (OUT1, ">contig.leftseq.fa")\|\|die "cannot open $ARGV[0]\n";  open (OUT2, ">contig.rightseq.fa")\|\|die "cannot open $ARGV[0]\n";  my $right = '';  my $left = '';  my $name = '';  my $len = $ARGV[1];  local $/ = '>';  while (<FILE>){  chomp;  my $head = '';  my $seq = '';  ($head,$seq) = split(/\n/,$_,2);  if ($head =~ /^(\S+)/){  $name = $1;  $seq =~ s/\n//g;  $seq = uc($seq);  $left = substr($seq,0,$len);  $right = substr($seq,-($len+1));  #$right = reverse $right;  print OUT1 ">$name\_left\n$left\n";  print OUT2 ">$name\_right\n$right\n";  }  }  close (FILE); |
| *fasta_manipulate.PEheader.pl* | #! user/bin/perl  #Zhuofei Xu, 2013-04-03  die "Usage: perl $0 fasta.input > out\n" unless (@ARGV == 1);  open (FILE, "$ARGV[0]")\|\|die "cannot open $ARGV[0]\n";  local $/ = '>';  my $count = 0;  my $len = 0;  my $name = '';  my $head = '';  my $sequence = '';  while (<FILE>){  chomp;  ($head,$sequence) = split(/\n/,$_,2);  if($head eq '')  {next;  } else{  $head =~ s/\s/:/;  $sequence =~ s/\n//g;  $len = length($sequence);  print ">$head:$len\n$sequence\n";  }  }  close (FILE); |
| *parse_PEread.1.pl* | #!/usr/bin/perl  use strict;  #Zhuofei Xu, April 03, 2013  die "Usage: perl $0 xaa2contigleft.blastn > matched.read.list \n" if (@ARGV != 1);  open(LIST,$ARGV[0]) or die "cannot open $ARGV[0]\n";  my $count = 0;  my $len = 0;  while(<LIST>){  chomp;  $len = 0;  if(/^(\S+)\s+(\S+)\s+(\S+)\s+(\S+)\s+(\S+)\s+(\S+)\s+(\S+)\s+(\S+)/){  my $name = $1;  my $identity = $3;  my $qstart = $7;  my $qends = $8;  if($name =~ /:(\d+)$/){  $len = $1;  }  my $hitlen = ($qends - $qstart + 1);  my $cover = $hitlen/$len;  if (($identity == 100) && ($hitlen >= 90) && ($cover >= 0.99)){  print "$_\n";  }  }  }  close LIST; |
| parse_PEread.2.pl | #!/usr/bin/perl  use strict;  #Zhuofei Xu, Sep 11, 2012  die "Usage: perl $0 hitread2left.tab > hitread2left.reverse.tab \n" if (@ARGV != 1);  open(LIST,$ARGV[0]) or die "cannot open $ARGV[0]\n";  my $count = 0;  while(<LIST>){  chomp;  if(/^(\S+)\s+(\S+)\s+(\S+)\s+(\S+)\s+(\S+)\s+(\S+)\s+(\S+)\s+(\S+)\s+(\S+)\s+(\S+)/){  my $sstart = $9;  my $sends = $10;  my $hitlen = ($sends - $sstart);  if ($hitlen < 0){  print "$_\n";  }  }  }  close LIST; |
| *parse_PEread.3.pl* | #!/usr/bin/perl  use strict;  #Zhuofei Xu, Sep 11, 2012  die "Usage: perl $0 hitread2left.tab > hitread2left.reverse.tab \n" if (@ARGV != 1);  open(LIST,$ARGV[0]) or die "cannot open $ARGV[0]\n";  my $count = 0;  while(<LIST>){  chomp;  if(/^(\S+)\s+(\S+)\s+(\S+)\s+(\S+)\s+(\S+)\s+(\S+)\s+(\S+)\s+(\S+)\s+(\S+)\s+(\S+)/){  my $sstart = $9;  my $sends = $10;  my $hitlen = ($sends - $sstart);  if ($hitlen < 0){  print "$_\n";  }  }  }  close LIST;  cat: cat: No such file or directory  #!/usr/bin/perl  use strict;  #Zhuofei Xu, April 04, 2013  die "Usage: perl $0 hitread2left.reverse.tab hitread2right.tab" if (@ARGV != 2);  open(LIST,$ARGV[0]) or die "cannot open $ARGV[0]\n";  open(FILE,$ARGV[1]) or die "can't open $ARGV[1]\n";  open (OUT1, ">circular.PE.list")\|\|die "cannot open $ARGV[0]\n";  open (OUT2, ">circular.PE.read.list")\|\|die "cannot open $ARGV[0]\n";  my $count = 0;  my %hash = ();  my %vash = ();  #HWI-ST897:147:C0MBGACXX:7:1315:8102:91659:2:N:0:CGTAGT:98 Contig00444_left 100.00 98 0 0 1 98 202 105 2e-51 194  #HWI-ST897:147:C0MBGACXX:7:1315:8102:91659:1:N:0:CGTAGT:98 Contig00444_right 100.00 98 0 0 1 98 358 455 2e-51 194  while(<LIST>){  chomp;  if(/^(\S+)\s+(\S+)\_\S+\s+(\S+)\s+(\S+)\s+(\S+)\s+(\S+)\s+(\S+)\s+(\S+)\s+(\S+)\s+(\S+)/){  my $name1 = $1;  my $contig1 = $2;  if ($name1 =~ /^(.*?):(\d):N:/){  $vash{$1} = $2;  push @{$hash{$contig1}}, "$1";  #warn "$contig1 $1 $2\n";  }  }  }  close LIST;  while(<FILE>){  chomp;  if(/^(\S+)\s+(\S+)\_\S+\s+(\S+)\s+(\S+)\s+(\S+)\s+(\S+)\s+(\S+)\s+(\S+)\s+(\S+)\s+(\S+)/){  my $name2 = $1;  my $contig2 = $2;  if ($name2 =~ /^(.*?):(\d):N:/){  my $label = $1;  my $pair = $2;  #warn "$label $pair\n";  if(exists $hash{$contig2}){  foreach my $ele (@{$hash{$contig2}}){  if($label eq $ele){  if($vash{$ele} ne $pair){  print OUT1 "$contig2\n";  print OUT2 "$contig2\t$label:$pair\t$ele".":$vash{$ele}\n";  }  }  }  }  }  }  }  close FILE; |
| intesection.pl | #! /usr/bin/perl -w  use strict;  open (DATA, $ARGV[0])\|\|die "Can't open file $ARGV[0]:$!\n";  open (FILE, $ARGV[1])\|\|die "Can't open file $ARGV[1]:$!\n";  my %hash = ();  while (<DATA>){  chomp;  if (/^>(\S+)$/){  $hash{$1} = $1;  }  }  close (DATA);  while (<FILE>){  chomp;  if (/^(\S+)/){  my $name = $1;  if (exists $hash{$name}){  print "$_\n";  }  }  }  close (FILE); |
| *fasta_manipulate.pl* | #! user/bin/perl  #Zhuofei Xu, 2012-06-19  die "Usage: perl $0 fasta.input (sequence in multi-line) > out\n" unless (@ARGV == 1);  open (FILE, "$ARGV[0]")\|\|die "cannot open $ARGV[0]\n";  my $right = '';  my $left = '';  my $name = '';  local $/ = '>';  while (<FILE>){  chomp;  my $head = '';  my $seq = '';  #warn "$_\n";  ($head,$seq) = split(/\n/,$_,2);  if ($head =~ /^(\S+)\s+/){  $name = $1;  }  $seq =~ s/\n//g;  $seq = uc($seq);  $left = substr($seq,0,100);  $right = substr($seq,-100);  #$right = reverse $right;  #warn "$seq\n$right\n$left\n";  unless($seq eq ''){  print ">$name\_concatenate200bp\n$right","$left\n";  #print ">$name\_right100bp\n$right\n";  }  }  close (FILE); |
| *extractHit454.1.pl* | #! user/bin/perl -w  use strict;  #Zhuofei Xu, 2012-06-20  die "Usage: perl $0 blastn.out > out\n" unless (@ARGV == 1);  open (FILE, "$ARGV[0]")\|\|die "cannot open $ARGV[0]\n";  my $identity = 0;  my $alnlen = 0;  my $gap = 0;  while (<FILE>){  chomp;  if (/^(\S+)\s+(\S+)\s+(\S+)\s+(\S+)\s+(\S+)\s+(\S+)\s+/){  $identity = $3;  $alnlen = $4;  $gap = $6;  if(($identity >= 99) && ($alnlen == 200) && ($gap == 0)){  print "$_\n";  }  }  }  close (FILE); |
| *extractHit454.2.pl* | #! user/bin/perl -w  use strict;  #Zhuofei Xu, 2012-06-20  die "Usage: perl $0 hit.out > plasmid.list\n" unless (@ARGV == 1);  open (FILE, "$ARGV[0]")\|\|die "cannot open $ARGV[0]\n";  my $contig = '';  my %hash = ();  print "ContigID\tNumber_of_reads_cover_end\n";  while (<FILE>){  chomp;  if (/^(\S+)\s+(\S+)\_concatenate/){  $contig = $2;  $hash{$contig}++;  }  }  close (FILE);  for my $tag (sort {$a cmp $b} keys %hash){  print "$tag\t$hash{$tag}\n";  } |
|  |  |
|  |  |
