# Supplementary material for: Hundreds of Circular Novel Plasmids and DNA Elements Identified in a Rat Cecum Metamobilome
Source: PLoS One. 2014 Feb 4;9(2):e87924. doi: 10.1371/journal.pone.0087924 (PMC3913684; doi:10.1371/journal.pone.0087924)
Supplement: Information S2 — Estimation of chromosomal contamination and Sequencing output composition. (DOCX) [file pone.0087924.s004.docx]

**Supporting Information S2. Estimation of chromosomal contamination and Sequencing output composition.**

Only few previous studies (e.g. Sentchilo et al., 2013) in mobilomics have identified complete, circular sequences and thus analysis has largely been based on linear fragments resembling plasmids (Kav et al., 2012; Ma et al., 2012; Zhang et al., 2011; Sentchilo et al., 2013). This makes contamination of samples with chromosomal DNA an important factor as it is difficult to ensure that a contig does not originate on a chromosome but on an extrachromosomal genetic element.

Even if chromosomal contamination is not an important factor in this study of complete circular elements, we had an interest in determining the mobilome purity to evaluate the success of the method in obtaining a sample free of chromosomal contamination. We used Meta-RNA (Huang et al., 2009) to detect reads mapping to rDNA in metagenomic samples, as a measure of chromosomal DNA. To calculate this fraction, we assumed that the average bacterium in the sample have a genome size of 5*10^6^  nt and 4 copies of 16s rDNA (average of all entries in rrnDB, <http://rrndb.umms.med.umich.edu/> , 09-09-2013). Taking into account the length of 16s rDNA of ca 1500 nt, the percentage of the genome constituted by 16s rDNA was calculated:  (4*1,500/5,000,000)*100=0.12%. Thus, 0.12% of reads in an entirely chromosomal sample should map to 16s rDNA, whereas 0% of reads from a mobilome sample should map to 16s rDNA. As seen in Table 1A, only few reads did map to bacterial 16s rDNA from either of the sequencing platform; 0.000723% (2 reads) from the 454 platform and 0.00338% (5407 reads) from the Illumina platform. This is equivalent to a sample purity of 99.4% (454) and 97.2% (Illumina). In combination with the prokaryotic chromosomal contamination determined by qPCR (1.2% compared to a *Pseudomonas putida* KT2440 control) (data not shown), we conclude that the sample is almost free of chromosomal contamination.

It is important to calculate the purity of the mobilome as previous studies have struggled to produce pure plasmid/mobilome samples. Further, the method of estimation used in this study  is independent of primer efficiency bias, as opposed to the method of estimation used in previous studies; PCR with universal 16s rDNA primers (Kav et al., 2012; Zhang et al., 2011). One previous study did note the number of reads mapping to 16s rDNA, but did not use this information to estimate the sample purity (Sentchilo et al., 2013).

Even though 5407 reads from the Illumina mapped to the 16s database, it is not sensible to make phylogenetic statements as the reads are too short for standard methods (Caporaso et al., 2010). By BLAST search, we have found that 0.15% of reads from the Illumina platform have hits in the phage database (identity > 95%, hit length > 79 nt). Also, 1 and 564 reads from the 454 and Illumina platforms, respectively, were mapped to the eukaryotic 18S rDNA database (same criteria, Table 1A). Considering that rat or human DNA are a likely source of eukaryotic contamination, and that mammals have hundreds of copies of the 18S rRNA gene the genome (e.g. humans with ≈600 copies) (Stults et al., 2008). Also, eukaryotic organisms are not expected to harbour large quantities of small circular extrachromosomal elements. On this basis, we disregarded the found eukaryotic contamination as it is not expected to influence our analysis.

By BLAST search, 11.58% and 13.49% of reads from 454 and Illumina, respectively, were found to hit the NCBI plasmid database. This rather low percentage corresponds with the presumption that the plasmid database is not representative for plasmids in the environment or the mobilome used in this study (Zhang et al., 2011; Ma et al., 2012).

| A   \| Sample Name \| 454  reads before filtering \| 454 reads after filtering \| Illumina  reads before filtering \| Illumina reads after filtering \| \| --- \| --- \| --- \| --- \| --- \| \| Number of reads \| 292,811 \| 276,656 (94.5% of reads) \| 161,902,848 \| 159,866,488 (98.7% of reads) \| \| Total nt \| 1.2*10^8^ \| 1.1*10^8^ \| 1.6*10^10^ \| 1.5*10^10^ \| \| mean  read length (nt) \| 417 \| 415 \| 98 \| 95 \| \| mean quality score \| 25 \| 25 \| 35 \| 36 \| \| Bacterial  16S rDNA \|  \| 2 (0.001%) \|  \| 5407 (0.003%) \| \| Eukaryotic 18S rDNA \|  \| 1 (0.000%) \|  \| 564 (0.000%) \| \| NCBI phage \|  \| 1  (0.000%) \|  \| 235256 (0.15%) \| \| NCBI plasmid \|  \| 31424 (11.58%) \|  \| 21573555 (13.49%) \|   B   \|  \| Number of  reads: \| N50 (nt) \| Max (nt) \| Mean (nt) \| Total (nt) \| Count \| \| --- \| --- \| --- \| --- \| --- \| --- \| --- \| \| Newbler \| 0.28M \| 2629 \| 8935 \| 1969 \| 7,03*10^5^ \| 357 \| \| IDBA-UD \| 160M \| 2803 \| 17294 \| 1839 \| 8,42*10^6^ \| 4575 \|   Table S6. A: Read statistics for platforms before and after filtering. Below, read hits to 16s rDNA, 18s rDNA, NCBI phage and NCBI plasmids databases. Identification of reads encoding 16S and 18S rDNA by Meta-RNA as well as reads BLASTing to phage and plasmid databases indicate the composition of the sequenced sample. B: Assembly statistics from Newbler (454) and IDBA-UD (Illumina) |
| --- | --- | --- | --- | --- | --- | --- | --- | --- | --- | --- | --- | --- | --- | --- | --- | --- | --- | --- | --- | --- | --- | --- | --- | --- | --- | --- | --- | --- | --- | --- | --- | --- | --- | --- | --- | --- | --- | --- | --- | --- | --- | --- | --- | --- | --- | --- | --- | --- | --- | --- | --- | --- | --- | --- | --- | --- | --- | --- | --- | --- | --- | --- | --- | --- | --- | --- |
