# Supplementary material for: Hundreds of Circular Novel Plasmids and DNA Elements Identified in a Rat Cecum Metamobilome
Source: PLoS One. 2014 Feb 4;9(2):e87924. doi: 10.1371/journal.pone.0087924 (PMC3913684; doi:10.1371/journal.pone.0087924)
Supplement: Information S3 — Rarefaction type evaluation of sequencing depth. (DOCX) [file pone.0087924.s005.docx]

**Supporting Information S3. Rarefaction type evaluation of sequencing depth.**

generating random subsets from interleaved paired end reads:

sed 'N;s/\n/_/' inputfile > output1 &                            ## replace every second newline with _  this merges SEQ_NAME and SEQ in fasta-file to one line from

sed 'N;s/\n/_/' output1 > output2 &                              ## again, replace every second newline with _. this will merge paired-end-readpairs on one line and still be resolved by replacing _ with newline

shuf -n <linenumber> output2 > output3 &                         ##randomly selects <linenumber> records (here 12,500,000/2; 25,000,000/2; 50,000,000/2; 75,000,000/2; 100,000,000/2; 125,000,000/2; 150,000,000/2)

sed 's/_/\n/g' output3 > final.fna &                            ## exchange _ for newline  from http://www.unix.com/shell-programming-scripting/190521-replacing-newline-character.html

assembling each subset:

nohup nice -n 19 idba_ud –r reads_in –o IDBA-UD_output_dir --pre_correction --num_threads 16 --min_contig 200 &

analyzing each subset

read_fasta -i IDBA-UD_output_dir/contig.fa|analyze assembly -x &           ## calculates longest contig, shortest contig, mean of contigs, total nt in contigs, number of contigs and N50.

results

| #reads (M) | 12,5 | 25 | 50 | 75 | 100 | 125 | 150 | 160 |
| --- | --- | --- | --- | --- | --- | --- | --- | --- |
| N50 | 970 | 747 | 1073 | 1464 | 1748 | 2023 | 2262 | 2333 |
| MAX | 10234 | 12494 | 12638 | 12638 | 15210 | 15186 | 17294 | 17294 |
| MIN | 200 | 200 | 200 | 200 | 200 | 200 | 200 | 200 |
| MEAN | 736 | 692 | 845 | 1014 | 1129 | 1187 | 1226 | 1241 |
| TOTAL | 2966293 | 5173044 | 7500590 | 8457063 | 8946043 | 9268275 | 9477553 | 9547866 |
| COUNT | 4030 | 7466 | 8869 | 8340 | 7920 | 7802 | 7727 | 7691 |
