# Supplementary material for: Hundreds of Circular Novel Plasmids and DNA Elements Identified in a Rat Cecum Metamobilome
Source: PLoS One. 2014 Feb 4;9(2):e87924. doi: 10.1371/journal.pone.0087924 (PMC3913684; doi:10.1371/journal.pone.0087924)
Supplement: Information S4 — Test of assembly of sequences with interspersed repeats using IDBA-UD. (DOCX) [file pone.0087924.s006.docx]

**Supporting Information S4. Test of assembly of sequences with interspersed repeats using IDBA-UD.**

To investigate how interspersed repeats influenced the assembly process for IDBA-UD, we created a set of random DNA sequences with repetitive elements of varying size. By in silico creation of an Illumina-type PE dataset, we were able to investigate the behaviour of the the *de novo* assembler IDBA-UD when encountering interspersed repeats. This test is particularly important as unfortunate placement of primers on repeated structures could influence the validity by creating false positive results in the *in vitro* confirmation of circularity  results in vitro. We found that IDBA-UD was unable to assemble sequences where the repeat was longer than a read. Further, none of the repeat sizes tested in this setup (50nt, 100nt, 250nt and 1000nt with a 1000nt sequence between them) resulted in contig structures that could be mistaken for circular elements in our detection of circularity. Thus, IDBA-UD broke contigs when encountering repeats of 100nt, 250nt and 1000nt, and only identical ends of up to 100 bases was found. No circular contigs were found from linear control sequences with any repeat size when running the two-step in silico circularity detection. On this basis, we conclude that false positive results from the workflow for detection of circular contigs of Illumina reads cannot result from interspersed repeats when IDBA-UD is used with the mentioned parameters. As many accessory elements are flanked by such interspersed repeats, complete assembly of plasmids with this structure is not possible,  leading to an underrepresentation of long sequences within the group of finished circular elements. We did not investigate the effect of interspersed repeats on assembly of 454 data.

*De novo* de Bruijn graph assembly is a *de facto* standard for Illumina reads in microbial metagenomics. The programs used for this have inherited problems with resolving repeats in the sequence, especially if the repetitive sequence is longer than the read. In theory, this can be resolved by paired end sequences, for repeats shorter than the read pair spans. By constructing  random DNA sequences and corresponding paired end reads (each 98 nt, insert 500 nt) with inserts with lengths of 50 nt, 100 nt, 250 nt, or 1000 nt, we wanted to test if the IDBA-UD assembler were able to resolve such structures. By BLASTing the sequences against the contigs created by the assemblers, I was able to determine that the assembler was not able to resolve repeats of 100 nt, 250 nt or 1000 nt while 50 nt repeats was resolved. For all repeats longer than the read  length, the assembler ‘break’ contigs when encountering a repeat, creating multiple contigs. This point to an inability of IDBA-UD assembler to correctly bridge repeats longer than 100 nt.

IDBA-UD takes into account uneven read depth of contigs, which is not an issue in this artificial case since depth is completely even. The assembler claim to make use of paired end information for scaffolding, the process of stitching together contigs if one read in a read-pair map on one contig and the other read of the same read pair map on another contig. By BLASTing the original, artificial sequences against the contigs, it was possible to graphically evaluate if the assembler had resolved the repeats correctly. Thus, if a repeat was resolved properly, a single contig the length of the original, artificial sequence would be seen whereas multiple contigs would be seen if the assembly was wrong or incomplete.

I created 100 random DNA sequences, each 1000 nt long from<http://www.bioinformatics.org/sms2/random_dna.html>.  (Naming of the sequences is sequential, starting with one and ending with 100). I then proceeded to scissor the random sequences together according to table 1. For the insert, the first 50 nt, 100 nt, 250 nt or 1000 nt were used. For each insert length, 3 independent sequences were created.  I have focused on long single repeats of either 50 nt, 100 nt, 250 nt or 1000 nt (figure 1).

| insert length   random sequence numbers(name of sequence)    length (nt) |
| --- |
| insert 1000 28,29,30,29,31  32,33,34,33,35  36,37,38,37,39      5000 |
| insert 250   40,41,42,41,43  44,45,46,45,47  48,49,50,49,51       3500 |
| insert 100   52,53,54,53,55  56,57,58,57,59  60,61,62,61,63       3200 |
| insert 50    64,65,66,65,67  68,69,70,69,71  72,73,74,73,75      3100 |

Table 1. Overview of random DNA sequences and
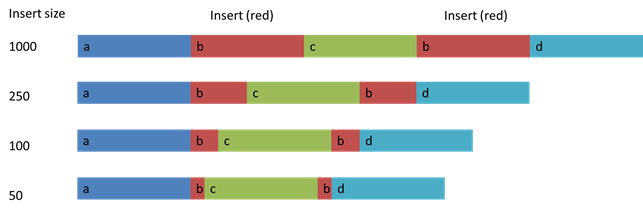
Figure 1. Schematic overview of artificial sequences.  Notice that ‘b’ is a single, long repeat of varying size and that the repeat is not inverse.

All bioinformatical processing was performed in a unix environment using Biopieces (Martin Asser Hansen, 2007), command line prompting and perlscripts found online.  Below is a detailed description of the making of paired end reads from a multi-fasta .fna file to complete interleaved paired end dataset. To mimic a ‘real life’ situation, artificial reads was mixed with 12.5M reads of a real paired end illumina dataset. The created reads are all 98 nt long and each artificial read is shifted exactly one nt to the right compared to the previous read. All read pairs are exactly 500 nt apart, making the spanable distance ca 700 nt.

Scripts and comments:

read_fasta -i random_fasta_in.fasta|write_fasta_files -k SEQ_NAME -d . -x    ##split entries into individual files

for x in *.fasta; do   mv -- "$x" "${x//,/_}"; done                          ##change , to _ in all file names in folder

for x in *.fasta; do read_fasta -i $x|split_seq -w 98|write_fasta -xo  $x.split; done   ##split sequences into 98base reads

cp *.split 2/                                                                   ##copy all entries to folder 2/ to manipulate (rev,comp, )

for x in *.split; do sed -e '1,1000d' $x > $x.2; done                        ## remove first 500 entries=first 500 bases resembling pair2 of paired end reads

rm *.split                                                                      ##remove files from before previous command

for i in *.2;do read_fasta -i $i|reverse_seq |complement_seq |write_fasta -xo $i.rc;done     ## reverse and complement entries

rm *.2                                                                          ##remove files from before previous command

../

for i in *.split;do read_fasta -i $i|write_tab -k SEQ_NAME -xo $i.names;done ##make lists of seq_names from read1

cd 2/                                                                           ##folder of pair2

for i in *.rc;do read_fasta -i $i|write_tab -k SEQ_NAME -xo $i.names;done    ##namelists of all file

(move names1 files to older names1/ and names2 files to names2/)

perl -e ' $separator="\t"; ($file1, $file2) = @ARGV; open (F1, $file1) or die; open (F2, $file2) or die; while (<F1>) { if (eof(F2)) { warn "WARNING: File $file2 ended early\n"; last } $line2 = <F2>; s/\r?\n//; print "$_$separator$line2" } if (! eof(F2)) { warn "WARNING: File $file1 ended early\n"; } warn "\nMerged $. lines side by side with separator $separator\nMerged files $file1 and $file2 side by side\n\n" ' ../names2/1_2_3_2X5.fasta.split.2.rc.names 1_2_3_2X5.fasta.split.names > names_test ## merge list2 and list1 into list2list1 tabular format, this is needed for changing the names of read2 to something to read1 (from http://sysbio.harvard.edu/csb/resources/computational/scriptome/unix/Tools/Merge.html)

ls *.rc > 1.txt                                                         ##creates a list with file names

sed 's/.2.rc//' 1.txt > 2.txt                                               ##remove end of file name, leaving common attribute for .fna and .tab pairs

for i in `cat 2.txt`;do read_fasta -i $i.2.rc|replace_vals -k SEQ_NAME -f $i.names.tab|write_fasta -xo $i.replacenames.fna;done              ##for each pair2 filepair .2.rc (fna) and .names.tab, exchange SEQ_NAME to match the ones from pair1

mv *.replacenames.fna ../                                               ##move fasta of pair2 files with new names to folder with pair1 files

for i in *.replacenames.fna;do sed 's/]/] 2:N:0:TAGCTG/' $i > $i.pair2;done ##change name of entries so that order_pairs can figure our that it is pair2

for i in *.split;do sed 's/]/] 1:N:0:TAGCTG/' $i > $i.pair1;done             ##change name of entries so that order_pairs can figure our that it is pair1

mkdir pair

mv *.pair1 *.pair2 pair/                                                    ##move pair1 and pair2 files prior to order_pairs (interleave)

ls *.pair1 > 1.txt ##creates a list with file names

sed 's/.pair1//' 1.txt > 2.txt   ##remove end of file name, leaving common attribute for pair1 and pair2 pairs

for i in `cat 2.txt`;do read_fasta -i $i.pair1,$i.replacenames.fna.pair2|order_pairs|write_fasta -xo $i.ordered;done                 ##interleave pairs in induvidual fasta files

##copy 12.5Mreads file to .

cat 12.5Mreadsfile *.ordered > 12.5Mplus24test.fna                      ##create file with both real reads and artificial ones

nohup nice -n 19 idba_ud -r 12.5Mplus24test.fna -o idba_ud --pre_correction --num_threads 16 --min_contig 200 &         ##assemble reads to contigs

The resulting contigs was used as BLAST search database for the 3*4 sequences seen in table 1 (Altschul et al., 1997).  All BLASTing, visualization and analysis was performed in CLC main workbench(6.7.1) with standard parameters.

I tested the correctness of interleaved reads by importing into ‘CLC genomics workbench’ where all read pairs aligned corretly to sequences. Thus, I conclude that the script for producing the artificial reads produced a realistic dataset.

Results:

1000 nt repeat:

For sequences with a single long repeat of 1000 nt:  in all three replicates, the sequence  is broken into 4 contigs, corresponding to a,b,c and d in figure 1. An overlap of 79 nt is seen between the contigs corresponding to a-b, b-c, c-b and b,d. This overlap in completely within the repeated sequence.

If the assembler was able to assemble the sequence correctly, a single contig would have been the result. However, since the repeat is longer than the read-pair (1000 nt vs. 700 nt), this result is expected.

In all cases, the BLAST hit of contigs represent the complete contig and not only a part of it. This is important as no sequence has been assembled wrongfully, only incomplete.
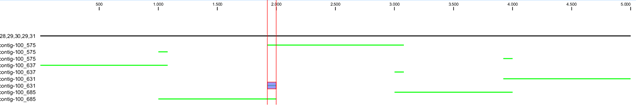
Figure 2. IDBA-UD contigs BLAST results of sequence with 1000bp repeat. Notice that the contig name for the contigs covering position 1000 to 2000 and 3000 to 4000 are identical, confirming that the two repeats are merged into one.

250 nt repeat:

It was expected that IDBA-UD could assemble the 250 nt repeats correctly into one single contig by use of scaffolding to bridge contigs (the repeat is much smaller than a read-pair spans). In contrast to this, IDBA-UD broke the sequence up into contigs corresponding to figure1; a,c and d but do not output the 250 nt repeat as a contig. Again, 79 nt overlaps are seen between a-c and c-d (figure 3).


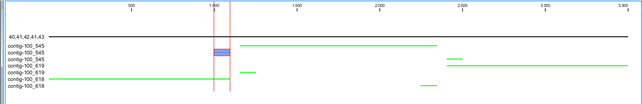
Figure 3. IDBA-UD contigs BLAST results of sequence with 250 bp repeat.

100 bp repeat

This repeat size is 2 nt longer than individual reads and thus, it is not possible to assemble the sequence correctly if reads are considered single end. Paired end reads should resolve the repeat with scaffolding. All three replicates assemble alike: The contigs corresponds to figure 1 a,b,c and d the following way: a+79nt of b, c with 79nt of b in either end, and d with 79 nt of b in the beginning (figure 4). Surprisingly, a 54 nt pair overlap is seen between a-c and c-d.


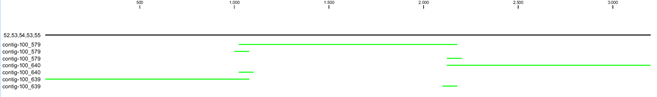
Figure 4. IDBA-UD contigs BLAST result of sequence with 100 nt single repeat.

50 nt repeat

where the 1000 nt repeat functions as a type of negative control because if is insolvable, a 50 nt repeat functions as a positive control: paired end information is not needed to resolve this repeat since the repeat is bridged by a single, 98 nt read. As seen in figure 5, IDBA-UD resolved this, as expected.
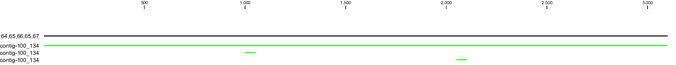
Figure 5. IDBA-UD contigs BLAST result of a sequence with a 50 nt single repeat. A single contig spans the entire sequence, showing that the repeat has been resolved properly.

Conclusion

Neither assembler succeeded in closing a repeat of the type and length tested here, by use of paired end information. This is surprising since both claims to use this information to connect contigs. Only for repeats shorter that individual reads, the assembler solved the sequence. This would presumably be possible for a single end dataset also, so paired end information does not have to be used.

No significant (e-value > 10^-5^) wrongful assembly is seen as this would have shown up in the BLAST-search. This is an important point in that it points to an unwillingness of the assemblers to output possible sequences if it is unclear if the sequences are true. At least in the case tested, false positives (wrongful assembly) are not existing while false negatives (incomplete, but solvable) are abundant.

Reference List

Altschul,S.F., Madden,T.L., Sch+ñffer,A.A., Zhang,J., Zhang,Z., Miller,W., and Lipman,D.J. (1997). Gapped BLAST and PSI-BLAST: a new generation of protein database search programs. Nucleic Acids Research *25*, 3389-3402.

Earl,D., Bradnam,K., John,J.S., Darling,A., Lin,D., Fass,J., Yu,H.O.K., Buffalo,V., Zerbino,D.R., and Diekhans,M. (2011). Assemblathon 1: A competitive assessment of de novo short read assembly methods. Genome Research *21*, 2224-2241.

Martin Asser Hansen. Biopieces.  2007.

Ref Type: Computer Program

Olorunniji,F. and Stark,W.M. (2010). Catalysis of site-specific recombination by Tn3 resolvase. Biochemical Society Transactions *38*, 417.

Peng,Y., Leung,H., Yiu,S.M., and Chin,F.Y.L. (2012). IDBA-UD: a de novo assembler for single-cell and metagenomic sequencing data with highly uneven depth. Bioinformatics *28*, 1420-1428.

Zerbino,D.R. and Birney,E. (2008). Velvet: algorithms for de novo short read assembly using de Bruijn graphs. Genome Research *18*, 821-829.
